# Tuning the Force, Speed, and Efficiency of an Autonomous Chemically Fueled Information Ratchet

**DOI:** 10.1021/jacs.2c07633

**Published:** 2022-09-08

**Authors:** Stefan Borsley, David A. Leigh, Benjamin M. W. Roberts, Iñigo J. Vitorica-Yrezabal

**Affiliations:** † Department of Chemistry, University of Manchester, Oxford Road, Manchester M13 9PL, U.K.; ‡ School of Chemistry and Molecular Engineering, East China Normal University, Shanghai 200062, China

## Abstract

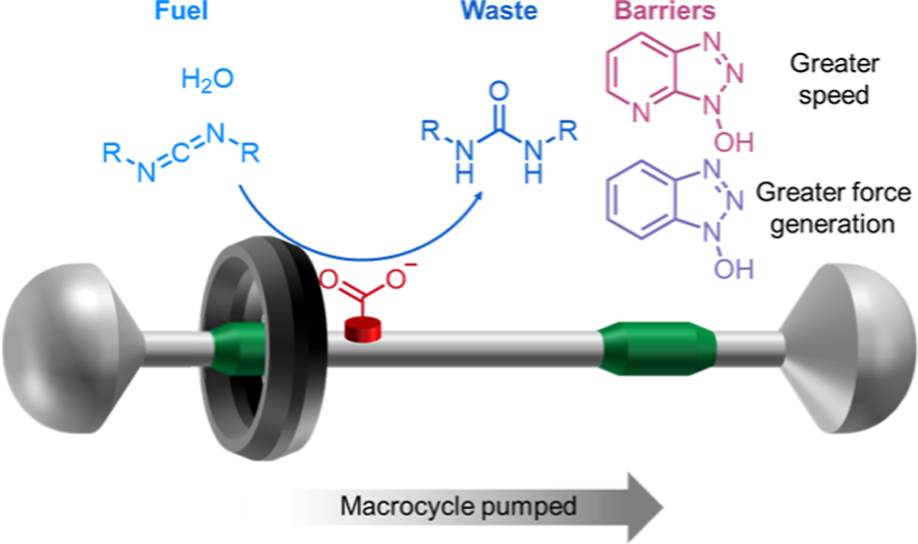

Autonomous chemically fueled molecular machines that function through information ratchet mechanisms underpin the nonequilibrium processes that sustain life. These biomolecular motors have evolved to be well-suited to the tasks they perform. Synthetic systems that function through similar mechanisms have recently been developed, and their minimalist structures enable the influence of structural changes on machine performance to be assessed. Here, we probe the effect of changes in the fuel and barrier-forming species on the nonequilibrium operation of a carbodiimide-fueled rotaxane-based information ratchet. We examine the machine’s ability to catalyze the fuel-to-waste reaction and harness energy from it to drive directional displacement of the macrocycle. These characteristics are intrinsically linked to the speed, force, power, and efficiency of the ratchet output. We find that, just as for biomolecular motors and macroscopic machinery, optimization of one feature (such as speed) can compromise other features (such as the force that can be generated by the ratchet). Balancing speed, power, efficiency, and directionality will likely prove important when developing artificial molecular motors for particular applications.

## Introduction

Autonomous chemically fueled information ratchets^1,2^ have recently been used as engines^3^ to drive synthetic molecular motors^4,5^ and pumps.^6−10^ Given the ubiquity of such processes in biology,^11^ understanding how to maximize their performance is important for synthetic molecular nanotechnology^12−18^ and artificial chemical fueling systems.^19−22^ With macroscopic machinery, optimizing one aspect of performance often compromises others. For example, diesel engines need to produce substantial torque to pull heavy loads, while gasoline engines are generally designed to maximize speed and/or efficiency.^23^ Features of biomolecular machines have evolved to similarly suit their functions:^24−27^ to transport cargo,^28,29^ a motor needs to be fast to beat diffusion, while the external force it can successfully pull against is less important. Conversely, speed of operation is less consequential for motor proteins in muscle cells,^11,30^ while the force they can exert is the primary requirement. Often trade-offs come from prioritizing a particular function:^24−29,31^ for example, for a macroscopic heat engine, conditions that give the maximum power output cannot also result in the most efficient operation.^32^

Distinct performance characteristics of molecular ratchets include speed, power, and efficiency. For autonomous chemically fueled information ratchets,^4−7^ these features relate to the directionality achieved and to the rate and efficiency of catalysis of the fuel-to-waste reaction by the ratchet.^33−36^ Appreciating how to manipulate these features and understanding the connections between them should help in optimizing molecular ratchet designs for specific tasks.

**Figure 1:**
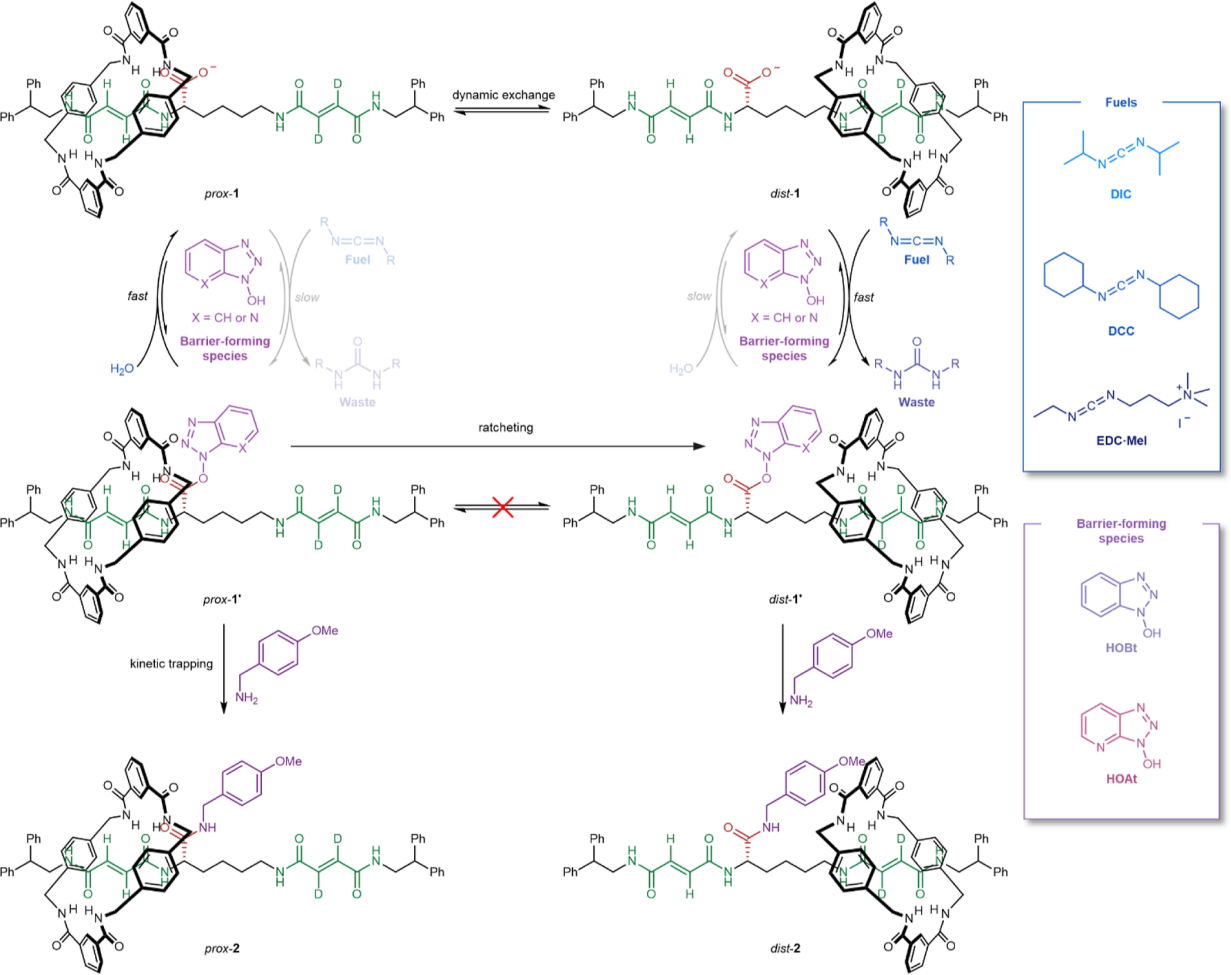
Chemomechanical cycle of carbodiimide-fueled molecular ratchet **1**. The macrocycle in **1** incessantly shuttles between two fumaramide binding sites (green), spaced non-equidistantly from either side of a carboxylate catalyst (red) on the axle. Ratchet **1** reacts with a carbodiimide fuel (blue) and a nucleophile (purple) to form a bulky activated ester, **1′**, in which the macrocycle is blocked from shuttling between the fumaramide sites. Hydrolysis of the ester under the fueling conditions regenerates **1**. Esterification occurs preferentially from co-conformer *dist*-**1** due to the steric clash between the fuel and the macrocycle, while activation of the ester by the hydrogen bonding from the macrocycle results in faster hydrolysis of *prox*-**1′** (disfavored, slower reaction pathways shown with faded arrows), resulting in pumping of the macrocycle to the distal fumaramide site. Equilibrium arrows are used for thermodynamic consistency (to obey microscopic reversibility), although under the experimental conditions, the backward steps are negligible. The reaction with *p*-methoxybenzylamine results in a kinetically stable amide as a permanent barrier, allowing the *dist*-**2**/*prox*-**2** ratio to be determined by ^1^H NMR spectroscopy.

The carboxylate-catalyzed hydration of carbodiimides has emerged as a robust, reliable, and versatile fuel-to-waste process for autonomously fueling molecular machines,^5,6^ paralleling its use in driving transient bond formation^37,38^ and dissipative assembly.^39−42^ The fuel cycle is particularly amenable for tuning in rotaxane-based information ratchets as the initially formed *O*-acyl urea can be displaced with a second nucleophilic catalyst^38−42^ that can act as a barrier-forming species^6^ (Figure 1). This allows the structure of both the fuel and the nucleophilic catalyst to be varied independently without affecting the structure of the carboxylate catalyst (here, a rotaxane ratchet).

Previously, it has been demonstrated that the distribution of the macrocycle between the two fumaramide sites in rotaxane ratchet **1** (Figure 1) can be driven away from the equilibrium by the hydration of diisopropylcarbodiimide (DIC).^6^ The DIC reacts with the carboxylic acid of the axle, transiently forming an *O*-acyl urea barrier to macrocycle shuttling. The O-acyl urea is quickly displaced by a nucleophilic catalyst [hydroxybenzotriazole (HOBt) or pyridine] to form a barrier that is hydrolyzed slowly on the time scale of macrocycle shuttling kinetics. Directional motion is generated through kinetic asymmetry^1,34−36^ in the chemical engine cycle.^3^ It arises from differences in rates of the machine co-conformers in both their reaction with DIC and in the hydrolysis of the barrier (the machine is doubly kinetically gated),^6^ the former being a classic Curtin–Hammett principle scenario.^3,43^

**Figure 2:**
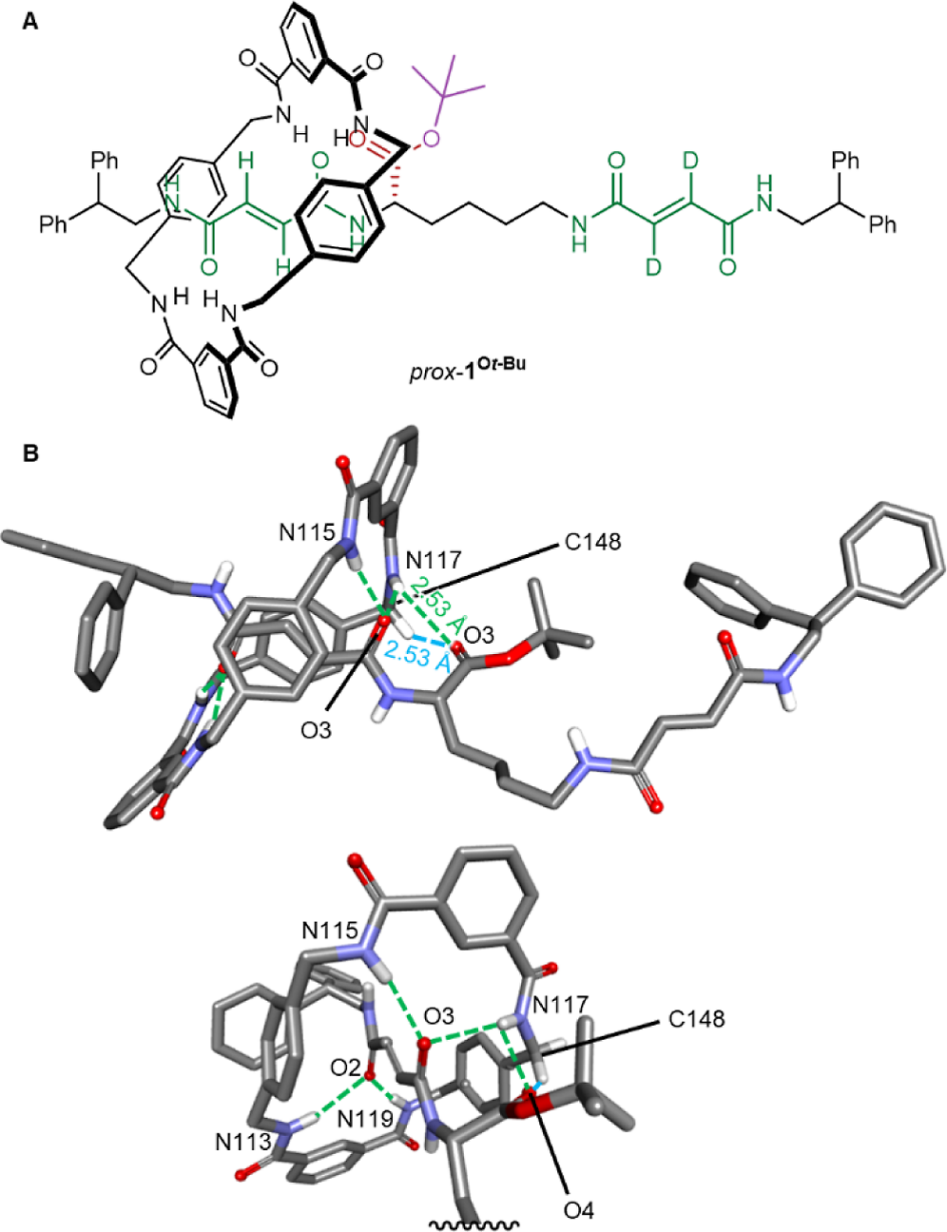
(A) Chemical structure of *prox*-**1**^**O*t*-Bu**^, the *t*-butyl ester of **1**. (B) X-ray crystal structure of *prox*-**1**^**O*t*-Bu**^. The *t*-butyl ester barrier is oriented away from the macrocycle. The upper far side of the macrocycle is puckered to form hydrogen bonds between an amide and benzylic protons of the macrocycle and the carbonyl oxygen of the thread ester group. Hydrogen bond lengths [Å]: O2···HN113, 2.36 Å; O2···HN119, 2.15 Å; O3···HN115, 2.07 Å; O3···HN117, 2.62 Å; O4···HN117, 2.53 Å; and O4···HC148, 2.53 Å. Hydrogen bond angles: O2···H–N113, 165.9°; O2···H–N119, 163.8°; O3···H–N115, 162.8°; O3···H–N117, 160.9°; O4···H–N117, 118.6°; and O4···H–C148, 126.8°. Hydrogen bonds between heteroatoms are shown in green, while CH hydrogen bonds are shown in blue. Solvate molecules and other hydrogen atoms are omitted for clarity.

**Figure 3:**
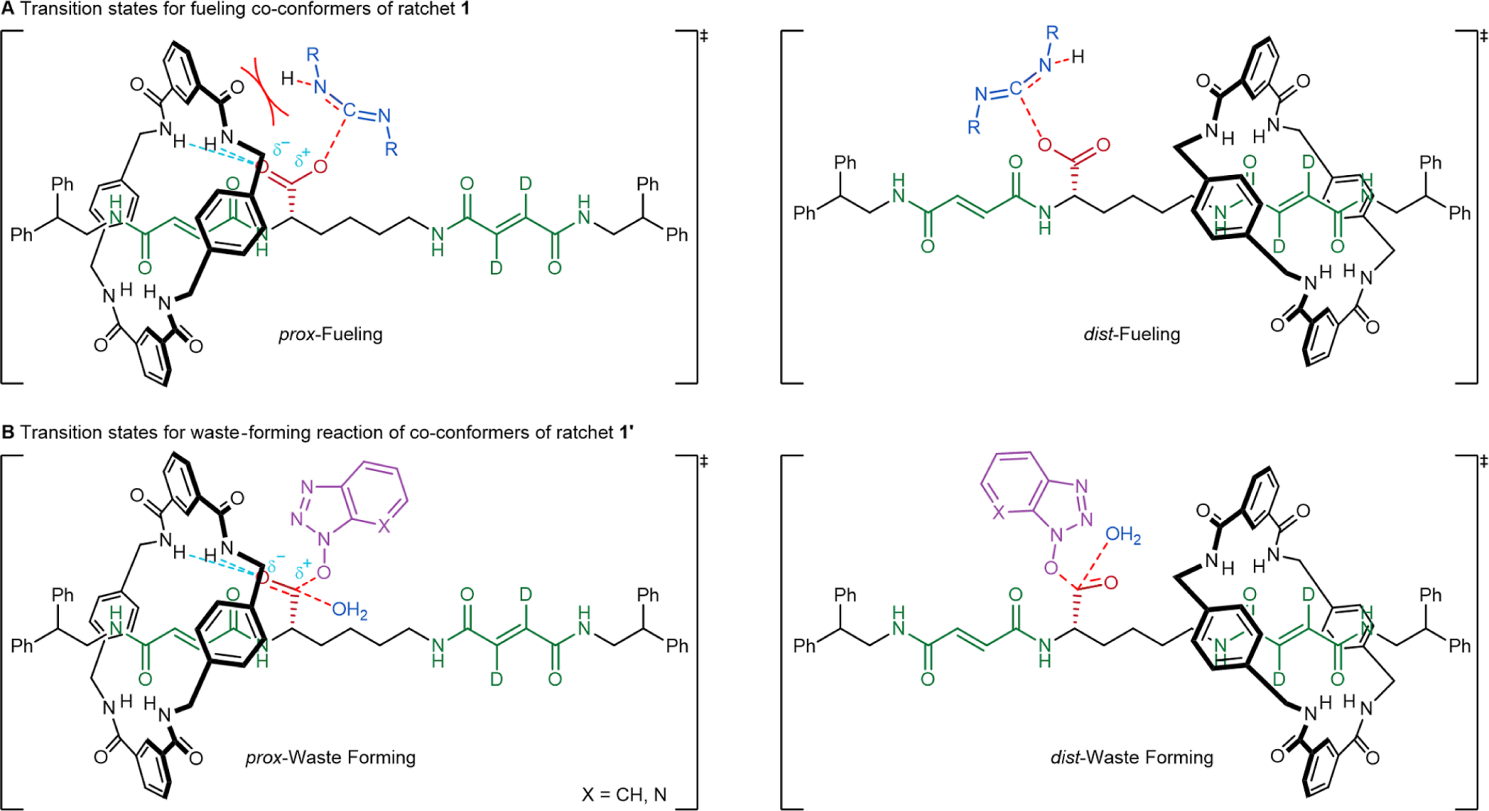
Proposed transition-state structure for (A) fueling and (B) waste-forming reactions for the *prox*- and *dist*- co-conformers of ratchet **1** with carbodiimide fuels and HOBt or HOAt. Formation/breaking of bonds in the transition state is shown as dotted red lines. Stabilizing interactions are shown as dotted blue lines. Steric hindrance disfavors fuel addition to *prox*-**1**, while stabilizing hydrogen bonding interactions with the macrocycle lowers the transition-state energy for the waste-forming reaction with *prox*-**1′**.

**Figure 4:**
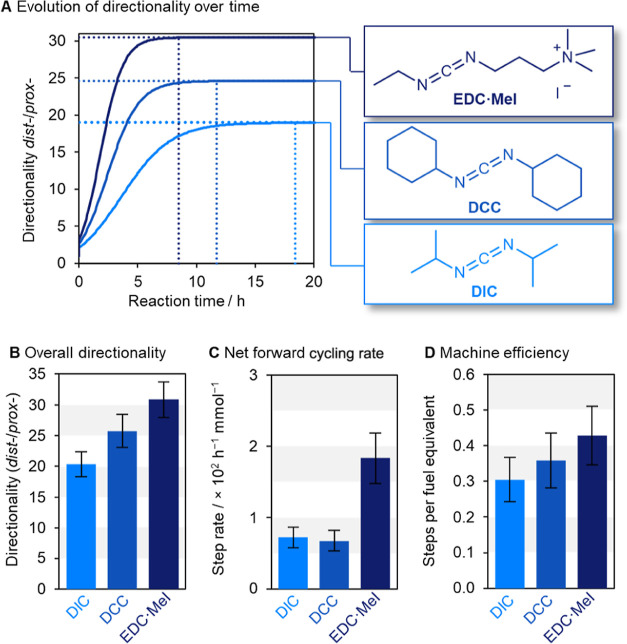
Operation of ratchet **1** with different carbodiimide fuels {[**1**] = 2.5 mM, [HOBt] = 5.0 mM, and [fuel] = 12.5 mM in MES-buffered (100 mM, pH_obs_ 5.36) MeCN-*d*_3_/H_2_O (7:3 *v*/*v*)}. (A) Graph plotting the directionality as a ratio of the distal and proximal isomers changing as the ratchet undergoes multiple cycles over time. (B) Maximum directionality reached by ratchet **1** under these conditions, with different carbodiimide fuels. (C) Net forward cycling rate of machine **1** with different carbodiimide fuels (see Supporting Information Section S5 for details). (D) Net forward steps per fuel equivalent, which corresponds to the overall efficiency of the machine as a percentage of the overall energy supplied by the fuel-to-waste reaction that is productively harnessed by machine **1**.^36^

**Figure 5:**
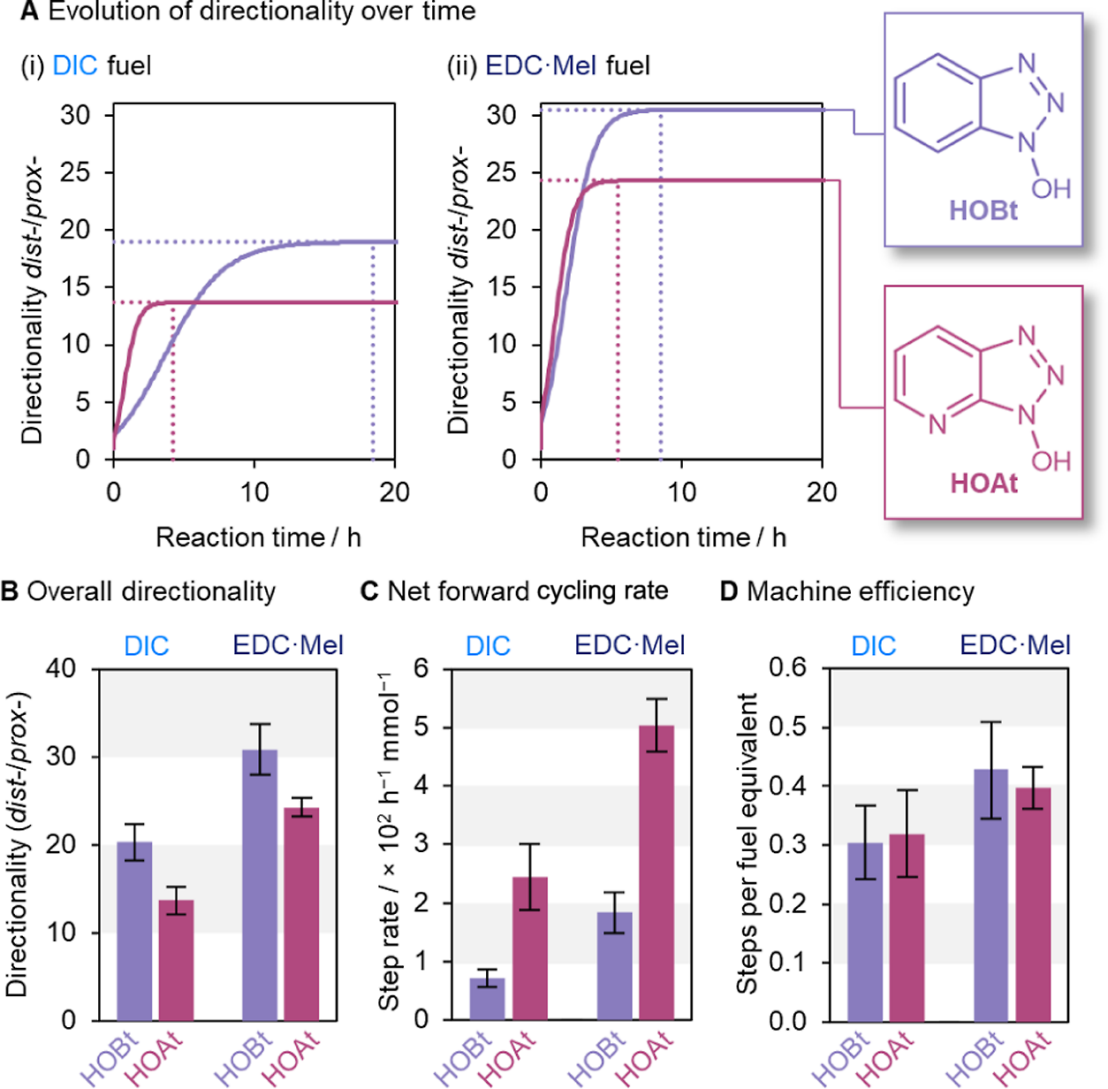
Operation of ratchet **1** as a function of varying the barrier-forming species for DIC and EDC·MeI fuels {[**1**] = 2.5 mM, [barrier-forming species] = 5.0 mM, and [fuel] = 12.5 mM in MES-buffered (100 mM, pH_obs_ 5.36) MeCN-*d*_3_/H_2_O (7:3 *v*/*v*)}. (A) Graph plotting the directionality as a ratio between the distal and proximal isomers as it changes over time with (i) DIC and (ii) EDC·MeI fuels. (B) Maximum directionality reached by machine **1** under these conditions, with different carbodiimide fuels. (C) Net forward cycling rate of machine **1** with different barrier-forming species (see Supporting Information Section S5 for details). (D) Net forward steps per fuel equivalent, which corresponds to the overall efficiency of the machine as a fraction of the overall energy supplied by the fuel-to-waste reaction that is productively harnessed by machine **1**.^36^

Here, we explore the relationships between the force, speed, power, and efficiency in molecular ratchet **1** (Figure 1). The X-ray crystal structure of **1** (Figure 2) shows structural features consistent with how kinetic asymmetry likely arises within the chemical engine cycle (Figure 3). We examine the effect of varying the structure of the fuel (Figure 4) and barrier (Figure 5) on the operation of the ratchet and consider the design implications for the development of future ratchets.

## Results and Discussion

### Relating Force, Speed, and Efficiency to the Operation of an Information Ratchet

Upon fueling with carbodiimide fuels, ratchet **1** performs work by pumping the macrocycle away from the equal distribution between the fumaramide sites on the axle at equilibrium in **1** to a nonequilibrium distribution in the **1**/**1′** mixture (Figure 1).^6^ The further the distribution is driven away from the equilibrium value, the more work the ratchet has to do to reach and maintain the out-of-equilibrium state. The ratio of *dist*-**1′**/*prox*-**1′** (where prime denotes any species where macrocycle shuttling between the fumaramide groups is blocked) in the steady state is a direct measure of the ratcheting constant, *K*_r_ (Supporting Information, Section S4.2),^34^ which quantifies kinetic asymmetry.^1,33^ Under these circumstances, with a negligible mechanical exchange between *prox*-**1′** and *dist*-**1′**, *K*_r_ quantifies the energy stored by the ratcheting process.^34,44^ In turn, the energy stored is related to the average force a ratchet can exert when pushing against a load, as force can be represented as a change in energy divided by distance. Qualitatively, the higher the energy stored by the ratchet (i.e., the higher the directionality), the higher the force that can be produced by its operation.^31^

The “speed” of the ratchet operation (i.e., the net forward chemomechanical cycle rate) is the rate of forward cycles that allow work to be done compared to the rate of backward cycles that undo that work and the rate of futile cycles that do no work but still consume fuel.^2,33,45^ This means that both the rate of catalysis and the directionality of the ratchet contribute to the speed of a motor. Likewise, the (potential) power of the ratchet is the force the ratchet produces (or could produce) at the net rate at which it proceeds forward around the chemomechanical cycle.^31^

“Machine efficiency” is the fraction of the available energy from the fuel-to-waste reaction that is harnessed productively by the machine, that is, the number of forward steps per unit fuel. Machine efficiency can be divided into “catalytic efficiency”^36^—the proportion of the fuel-to-waste reactions that proceed by the machine-catalyzed pathway—and “thermodynamic efficiency”^24−27,36,44^—the proportion of energy released from the fuel-to-waste reaction that is harnessed by the machine. In common with the force, power, and speed, the thermodynamic efficiency is linked to the kinetic asymmetry (and hence the directionality) of the operation.^31^

Understanding how structural features affect directionality and the rate and efficiency of fuel use by ratchets is therefore key for tuning the force, speed, power, and efficiency of a ratchet in driving nonequilibrium functions and processes.^46−49^

### X-ray Crystal Structure of Ratchet **1**

We previously found^6^ that fueling ratchet **1** with DIC in the presence of HOBt as a barrier-forming species drives the macrocycle distribution from the equilibrium value of 50:50 to 5:95 in favor of the distal co-conformer. The directionality arises from kinetic gating of both the fuel addition and barrier hydrolysis steps. This can be rationalized by the steric clash between the fuel and the macrocycle, resulting in a slower rate of fuel addition to co-conformer *prox*-**1** than to *dist*-**1**, while hydrogen bonding between the macrocycle and the carboxylate group results in faster barrier hydrolysis of *prox*-**1**^**OBt**^ than of *dist*-**1**^**OBt**^. We attempted X-ray crystallography on a range of derivatives of **1** to see if we could obtain solid-state structural evidence in support of either or both parts of this putative mechanism.

A single crystal of *prox*-**1**^**O*t*-Bu**^ (the *t*-butyl ester of ratchet **1**) suitable for X-ray diffraction was obtained by slow evaporation of a solution of rotaxane in CH_2_Cl_2_/MeOH (Figure 2 and Section S7, Supporting Information, CCDC deposition code: 2191007). The solid-state structure shows four NH···O hydrogen bonds between the amides of the macrocycle and the fumaramide carbonyls on the thread (average NH···O distance = 2.30 Å). The bulky *tert*-butyl barrier is oriented away from the macrocycle, reflective of the steric clash between the macrocycle and the barrier envisaged as being responsible for the difference in the rate of fuel addition between *dist*-**1** and the more sterically encumbered *prox*-**1** co-conformer (Figure 3a).

In the solid-state structure, hydrogen bonding is also apparent between the macrocycle and the carbonyl of the ester. The macrocycle is puckered, with the upper far side (as viewed in Figure 2B) angled toward the ester. Two hydrogen bonds are present between the macrocycle and the ester carbonyl, from the amide N–H (Figure 2B, green, NH···O distance = 2.53 Å, and angle = 118.6°) and from a benzylic C–H hydrogen atom (Figure 2B, blue, CH···O distance = 2.53 Å, and angle = 126.8°). Such hydrogen bonding would polarize the C=O bond of the ester.^50^ If present during the chemomechanical cycle in solution, this interaction would stabilize the developing negative charge^46^ at that oxygen atom of the proximal co-conformer in the transition state during barrier hydrolysis, leading to faster hydrolysis of *dist*-**1′** than of *prox*-**1′** (Figure 3B).

### Varying the Fuel Structure

With X-ray crystallography providing structural evidence in support of the origin of directional selectivity in the operation of information ratchet **1**, we next explored the effects of changing the structure of the fuel. The original DIC fuel was compared to two other common carbodiimide coupling agents:^51^ dicyclohexylcarbodiimide (DCC) and 1-ethyl-3-(3-dimethylaminopropyl)carbodiimide methyliodide (EDC·MeI)^52^ (Figure 4). Experiments were conducted using the conditions optimized for directionality^6^ {[**1**] = 2.5 mM, [barrier-forming species] = 5.0 mM, and [fuel] = 12.5 mM in 2-(*N*-morpholino)ethanesulfonic acid (MES)-buffered (100 mM, pH_obs_ 5.36) MeCN-*d*_3_/H_2_O (7:3 *v*/*v*), Section S2.1, Supporting Information}. These conditions ensure that the vast majority (approximately 95%) of rotaxane **1**/**1′** is present as **1′** while fuel remains during the reaction.^6^ The operating conditions were buffered to acidic conditions at which carbodiimide activation is effective^53^ with phosphate buffers avoided because of the known issue of phosphate-induced decomposition of carbodiimides.^54^ The rate of the fuel-to-waste reaction was monitored by ^1^H nuclear magnetic resonance (NMR) spectroscopy. The macrocycle distribution formed under out-of-equilibrium fueling was kinetically trapped at different stages by the addition of *p*-methoxybenzylamine (Figure 1, bottom), allowing the evolution of directionality to be determined by ^1^H NMR of the kinetically trapped species. The results of the experiments with the different fuels are shown in Table 1.

**Table 1:** Experimentally Determined Parameters for Directionality, Speed, and Efficiency of the Operation of Ratchet **1** with Different Fuel/Barrier Combinations {[**1**] = 2.5 mM, [Barrier-Forming Species] = 5.0 mM, and [Fuel] = 12.5 mM in MES-Buffered (100 mM, pH_obs_ 5.36) MeCN-*d*_3_/H_2_O (7:3 *v*/*v*)}

entry	fuel	barrier-forming species	overall directionality, *dist*-**1**/*prox*-**1**	fueling gating	waste-forming gating	net forward step rate/×10^2^ h^–1^ mM^–1^	overall efficiency^21^
1	DIC	HOBt	20.3 ± 2.0:1	2.2 ± 0.03	9.3 ± 0.9	0.72 ± 0.15	30% ± 6%
2	DCC	HOBt	25.7 ± 2.6:1	2.6 ± 0.04	9.4 ± 1.0	0.67 ± 0.15	36% ± 8%
3	EDC·MeI	HOBt	30.9 ± 2.9:1	3.1 ± 0.04	9.7 ± 0.9	1.83 ± 0.36	43% ± 8%
4	DIC	HOAt	13.7 ± 1.5:1	1.7 ± 0.04	8.0 ± 0.9	2.45 ± 0.56	32% ± 7%
5	EDC·MeI	HOAt	24.3 ± 1.1:1	3.5 ± 0.03	7.0 ± 0.3	5.03 ± 0.46	40% ± 4%

As evidenced by the directionality observed upon the addition of *p*-methoxybenzylamine at 0 h and modeling of the kinetics (Supporting Information, Section S4), only the gating of the fueling step is significantly affected by these variations in the fuel structure (Table 1). This confirms that neither the carbodiimide nor the formation of the urea waste product plays a role in the barrier removal step. Ratchet **1** gives better directionality with either DCC or EDC·MeI (Table 1, entries 2 and 3) than with DIC (Table 1, entry 1) (Figure 4A,B). DCC is bulkier than DIC, which exacerbates the steric clash when the macrocycle is on the proximal fumaramide site (Figure 3B). Steric issues may also contribute to the better fueling gating observed with EDC·MeI (Table 1, entry 3). However, the positive charge on the quaternary ammonium group of this fuel leads to faster reaction rates with carboxylic anions.^55^ This effect may be greater for *dist*-**1** than for *prox*-**1**, if hydrogen bonding similar to that observed with the ester in Figure 2 also polarizes the carboxylate. Both steric and electronic effects likely lead to EDC·MeI providing the best fueling gating of the three carbodiimide fuels investigated.

Directionality is not the only important factor to consider in molecular ratchet design.^24−29,31^ The rate of the machine-catalyzed fuel-to-waste reaction determines how fast a motor can operate.^1,31,33,36^ Here also, EDC·MeI was the preferable carbodiimide fuel as it reacts faster, which combined with the better directionality means that the ratchet undergoes net forward chemomechanical cycles at over double the rate with EDC·MeI compared to that with DIC or DCC (Figure 4C). Although DCC showed better directionality than DIC, they have a similar step rate due to the significantly slower reaction of DCC than that of DIC under these conditions.

Only very modest changes were observed in the overall machine efficiency under the conditions optimized for directionality.^6^ EDC·MeI showed a marginally better catalytic efficiency (Supporting Information, Section S3), with 64% of the fuel-to-waste reactions proceeding via the machine-catalyzed pathway (compared to 52% and 54% for DIC and DCC, respectively). This improved catalytic efficiency and directionality result in the best overall efficiency for EDC·MeI (Figure 4D). However, errors propagated from the directionality measurements (Supporting Information, Section S6) make it difficult to assess the overall efficiency with a high degree of accuracy.

### Varying the Barrier Structure

Altering the structure of the barrier-forming species provides another opportunity to influence the operating cycle of the molecular ratchet (Figure 1). The operation of the machine under the optimized conditions^6^ (Section S2.1, Supporting Information) with different barrier-forming species, HOBt and hydroxyazabenzotriazole (HOAt), was examined in combination with both DIC and EDC·MeI. In line with the proposed mechanism of operation (Figure 1), varying the barrier structure was found to principally alter the gating of the ester hydrolysis step (Table 1).^56^

HOBt gave rise to a better overall directionality of the ratchet than HOAt (Figure 5A,B). However, the time at which the maximum directional bias (steady state) was reached was significantly decreased with HOAt. This trend was observed using either fuel (Figure 5A) but is most obvious using DIC: the maximum directionality was reached after 18 h with HOBt but 4× faster (4.5 h) with HOAt [Figure 5A(i)].

The differences in the directionality and rates can be rationalized from a consideration of the barrier structures. The activated ester **1**^**OAt**^ is significantly more hydrolytically labile than the **1**^**OBt**^ counterpart due to both the increased negative charge stabilization (making ^–^OAt a better leaving group) and hydrogen bonding to water via the pyridyl nitrogen (stabilizing the transition state for hydrolysis; Figure 3).^57^ Barrier hydrolysis is rate-limiting under these conditions, and therefore, faster hydrolysis results in a faster machine-catalyzed fuel-to-waste reaction, giving rise to faster cycling of the chemical engine^3^ and more rapid buildup of directional bias. The use of HOAt instead of HOBt increases the rate of cycling 3-fold with EDC·MeI fuel or 4-fold with DIC fuel (Figure 5C). The more stable transition state for the hydrolysis of **1**^**OAt**^ may also be responsible for the lower directionality obtained when using HOAt than that obtained using HOBt. The additional hydrogen bonding to water that stabilizes the transition state is likely to be present in both *prox*-**1**^**OAt**^ and *dist*-**1**^**OAt**^ and may compete with stabilizing hydrogen bonding from the macrocycle in *prox*-**1**^**OAt**^, resulting in reduced directionality.

While HOAt results in a faster fuel-to-waste reaction, HOAt also accelerates the background fuel-to-waste reaction (Supporting Information, Section S3.1), which reduces the catalytic efficiency of the molecular ratchet (Supporting Information, Section S3.1). Consequently, no significant differences are observed in the overall machine efficiency between HOBt and HOAt (Figure 5D).

### Tuning the Force, Speed, and Efficiency of Ratchet **1**

For the operation of ratchet **1**, EDC·MeI was found to be the best fuel in terms of directionality (Figure 4B), as well as the rate (Figure 4C) and efficiency (Figure 4D) with which it reacts with the ratchet. Consequently, fueling with EDC·MeI results in the highest force, speed, power, and efficiency of operation of ratchet **1**. Upon varying the barrier structure, the picture is more complex. HOBt gives better directionality, while HOAt enables faster catalyst engine cycles. As the force that can be generated is proportional to directionality, the use of HOBt would be the best for a function that required a high force output. By contrast, if speed was more important (e.g., perhaps for transport using a catenane motor^9^ based on ratchet **1**), HOAt would be more suitable as the faster rate of reaction results in faster net forward cycling despite lower directionality.

The changes between HOBt and HOAt illustrate not only the benefits but also the challenges in optimizing for a particular feature; improvements in one aspect of machine operation may lead to sacrifices in other properties. Different interactions with the solvent, varying amounts of the water content, waste production, and the pH may all influence different features of machine performance.^3,31^

## Conclusions

In addition to directionality, the characteristics of force, speed, and efficiency of a molecular ratchet (**1**) can be tuned with a judicious choice of the fuel and barrier structure. While the latter three features are sometimes maximized together (changing the fuel from DIC to EDC·MeI, for example), there may be trade-offs to be made if one trait is particularly desired. The increase in the speed of the engine cycle that arises from using HOAt instead of HOBt is at the expense of poorer directionality. Consequently, the speed of the ratchet is increased, while the potential force output is decreased. For some tasks, the time that a nonequilibrium steady state can be maintained for may also be important.^34−42,58^ For chemical engines based on **1**, this would be best served by using either DCC or DIC as the fuel, rather than EDC·MeI, as DCC and DIC react more slowly.

These kinds of trade-offs in aspects of performance are ubiquitous in the design of macro-scale machines and are also evident in the behavior of biological molecular machines.^12−17^ It seems very likely, therefore, that the ability to tune the structures of chemical engines and fuels to suit particular tasks will prove important in the development of artificial molecular nanotechnology.

## Supporting Information Available

Experimental methods and operations data and X-ray crystal structure determination method and data (PDF)

## Abbreviations

DCC: dicyclohexylcarbodiimide
DIC: diisopropylcarbodiimide
DMSO: dimethyl sulfoxide
EDC: 1-ethyl-3-(3-dimethylaminopropyl)carbodiimide
HOAt: hydroxyazabenzotriazole
HOBt: hydroxybenzotriazole
MES: 2-(*N*-morpholino)ethanesulfonic acid
NMR: nuclear magnetic resonance
