# Supplementary material for: Tuning the Force, Speed, and Efficiency of an Autonomous Chemically Fueled Information Ratchet
Source: J Am Chem Soc. 2022 Sep 8;144(37):17241–8. doi: 10.1021/jacs.2c07633 (PMC9501901; doi:10.1021/jacs.2c07633)
Supplement: Supplementary file 1 — ja2c07633_si_001.pdf [file ja2c07633_si_001.pdf]

Supplementary Information

Tuning the Force, Speed and Efficiency of an Autonomous Chemically Fueled Information Ratchet

Stefan Borsley,[1‡] David A. Leigh,*[1,2] Benjamin M. W. Roberts[1‡] and Iñigo J. Vitorica-Yrezabal[1]

[1]Department of Chemistry, University of Manchester, Oxford Road, Manchester M13 9PL, United Kingdom

[2]School of Chemistry and Molecular Engineering, East China Normal University, 200062 Shanghai, China

* david.leigh@manchester.ac.uk

‡ These authors contributed equally.

Contents

S1. General experimental procedures S3
S2. Experimental methods S4
S2.1 Operation conditions for measurement of kinetics and directionality S4
S2.1 Crystallization conditions S4
S3. Determination of pseudo-first order rate constants S5
S3.1 General method for determining machine catalyzed rate S8
S3.2 Determination of catalytic efficiencies S8
S4. Measurement of directionality S10
S4.1 Finding gating for ester formation and hydrolysis S14
S4.2 Equating directionality and the ratcheting constant (kinetic asymmetry) S15
S4.3 Fuel molecules used per forward step S17
S5. Net rate of forwards movement S18
S6. Comment on errors S19
S7. X-ray crystal structure information S20
S8. References S21

S1. General experimental procedures

Unless stated otherwise, reagents were obtained from commercial sources and used without purification. All chemicals, reagents, were purchased from Sigma Aldrich, UK (Merck KgaA) or Fluorochem UK. Deionized water was obtained by a milli-Q water purifier (Millipore). Anhydrous solvents were obtained by passing the solvent through an activated alumina column on a Phoenix SDS (solvent drying system; JC Meyer Solvent Systems, CA, USA). ^{1}H NMR spectra were recorded on a Bruker Avance III instrument with an Oxford AS600 magnet equipped with a cryoprobe [5 mm CPDCH ^{13}C-^{1}H/D] (600 MHz) at a constant temperature of 20 °C. ^{1}H and ^{13}C chemical shifts are reported in parts per million (ppm) from low to high field and referenced to the literature values for chemical shifts of residual non-deuterated solvent, with respect to tetramethylsilane (0.00 ppm) as an external standard. Standard abbreviations indicating multiplicity are used as follows: s (singlet), bs (broad singlet), d (doublet), t (triplet), dd (doublet of doublets), ddd (doublet of doublets of doublets), ddt (doublet of doublets of triplets), m (multiplet), *J* (coupling constant – quoted in Hz). All spectra were analyzed using MestReNova (Version 14.1.2). Fully characterized compounds were chromatographically homogeneous. Flash column chromatography was carried out using Silica 60 Å (particle size 40–63 μm, Sigma Aldrich, UK) as the stationary phase. TLC was performed on precoated silica gel plates (0.25 mm thick, 60 F254, Merck, Germany) and visualized using both short and long wave ultraviolet light in combination with standard laboratory stains (basic potassium permanganate, acidic ammonium molybdate and ninhydrin). Low resolution ESI mass spectrometry was performed with a Thermo Scientific LCQ Fleet Ion Trap Mass Spectrometer or an Agilent Technologies 1200 LC system with an Advion Expression LCMS single quadrupole MS detector.

Synthesis

Machine **1** was prepared according to reported procedures.[S1]

Buffer preparation and pH reporting

Buffer stocks were made at a 1 M concentration in H_2O or D_2O, as appropriate, and adjusted to the desired pH/pH$_{obs}$ by addition of HCl/DCl and NaOH/NaOD. The pH/pH$_{obs}$ values recorded for individual experiments are those of these 1 M buffer solutions before dilution with organic and aqueous solvent as required.

Abbreviations

DCC, dicyclohexylcarbodiiimide, DIC, diisipropylcarbodiimide; DMSO, dimethyl sulfoxide; EDC, 1-ethyl-3-(3-dimethylaminopropyl)carbodiimide; HOAt, hydroxyazabenzotriazole; HOBt, hydroxybenzotriazole; MES, 2-(*N*-morpholino)ethanesulfonic acid; NMR, nuclear magnetic resonance.

S2. Experimental methods

S2.1 Operation conditions for measurement of kinetics and directionality

Reaction conditions: [**1**] = 2.5 mM, [barrier-forming species] = 5.0 mM, [carbodiimide fuel] = 12.5 mM, [buffer] = 100 mM, solvent: CD_3CN/D_2O (7:3 *v*/*v*), pH_{obs} 5.36

Carbodiimide fuels: Diisopropylcarbodiimide (DIC), Dicyclohexylcarbodiimide (DCC), or 1-Ethyl-3-(3-dimethylaminopropyl)carbodiimide Methiodide (EDC·MeI)

Barrier-forming species: Hydroxybenzotriazole (HOBt) or Hydroxyazabenzotriazole (HOAt)

Ratchet **1** (2.54 mg, 1.25 mmol) was weighed into an NMR tube and dissolved in MES-buffered (100 mM, pH_{obs} 5.36) CD_3CN/D_2O (7:3 *v*/*v*) (0.5 mL). A solution of barrier-forming species (2.50 mmol, 12.5 mL of a 0.2 M solution in CD_3CN/D_2O, 1:1 *v*/*v*) was added. A solution of carbodiimide fuel (6.25 mmol, 6.25 mL of a 1 M solution in CD_3CN) was added and the reaction was monitored by ^1H NMR spectroscopy. The solution was left to react for the desired time at room temperature, after which time 4-methoxybenzylamine (12.5 mmol, 6.25 mL of a 0.5 M solution in CD_3CN) was added (for the 0 h experiment amine was added before DIC). The reaction mixture was left for a further 18 h then the mixture of amide rotaxanes *prox*-**2**/*dist*-**2** were fully precipitated from solution by addition of H_2O (2 mL). The mixture was filtered through cotton wool and washed with 1 M HCl (2 × 2 mL). The rotaxane was dissolved in MeOH and solvent was removed under reduced pressure. The residue was redissolved in DMSO-d_6 and a ^1H NMR spectrum was obtained, from which the ratio of *prox*-**2**:*dist*-**2** was determined.

Analogous conditions, but without the addition of ratchet **1**, were used to assess the rate of background (uncatalyzed) fuel use under the experimental conditions.

S2.1 Crystallization conditions

Rotaxane $\mathbf{1}^{\mathbf{O}t\text{-}\mathbf{Bu}}$ (~10 mg)[S1] was dissolved in a minimum of CH_2Cl_2/MeOH (9:1 *v*/*v*) and placed in a vial with a plastic cap. The cap of the vial was pierced a single time with a narrow-bore needle and placed inside a larger sealed vial containing MeOH, allowing the crystallization to occur via slow vapor diffusion.

S3. Determination of pseudo-first order rate constants

The ratio of ^1H NMR signals corresponding to the fuel and waste species was monitored throughout the course of the reaction left longest before quenching (typically 18 h). These were used to plot the concentrations of each species during the machine catalyzed or background reactions. The data were then used to fit pseudo-first order rate curve using non-linear regression, to determine a pseudo-first order rate constant and estimated starting concentration of fuel in each case (Figures S1–S5).

In machine catalyzed reactions, the fuel is used more quickly as the steady state distribution of the machine is reached and, as a result, only points from after the steady state is reached are considered during the fit. For this reason, the fit for the catalyzed data generally starts from a lower concentration than the uncatalyzed data. We have shown previously that this initial faster rate of fuel use is expected and can be accounted for in more complex kinetic models,[S1] however, these models were not necessary for the present study where a pseudo-fiarst order approximation suffices.

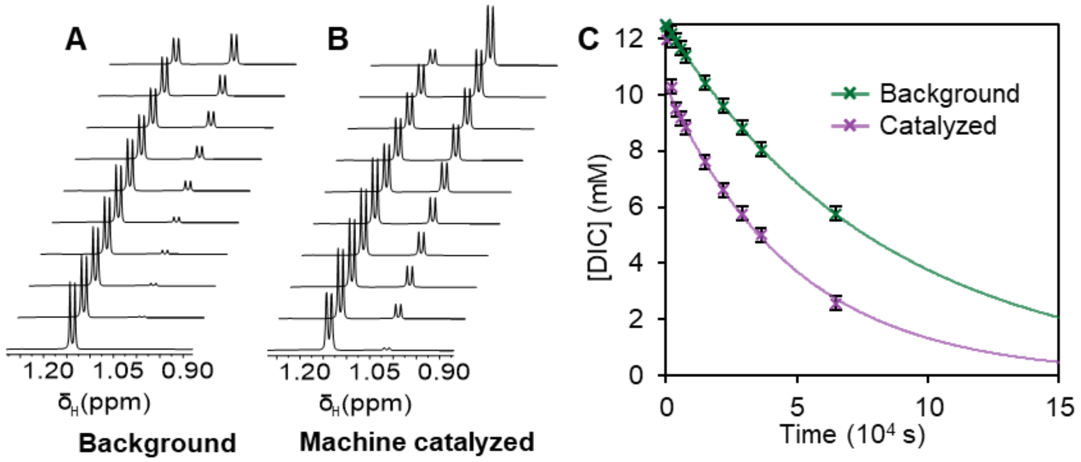

Figure S1 Kinetics of DIC hydration with a HOBt additive at pH_{obs} 5.36 in CD_3CN/D_2O (7:3 *v*/*v*). Stack-plots of partial ^1H NMR spectra following the background (**A**) and machine catalyzed (**B**) hydration of DIC. (**C**) The plotted data derived from the ^1H NMR spectra with the fitted pseudo-first order curves.

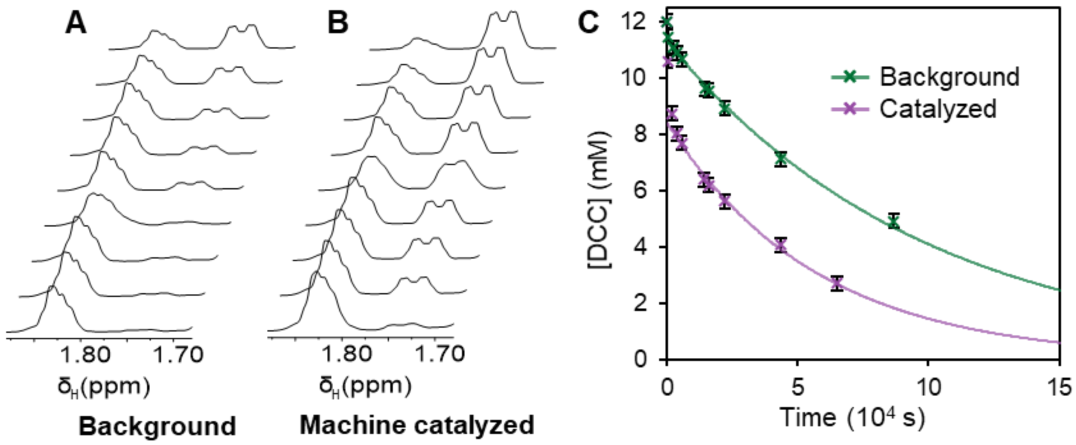

Figure S2 Kinetics of DCC hydration with a HOBt additive at pH_{obs} 5.36 in CD_3CN/D_2O (7:3 *v*/*v*). Stack-plots of partial ^1H NMR spectra following the background (**A**) and machine catalyzed (**B**) hydration of DCC. (**C**) The plotted data derived from the ^1H NMR spectra with the fitted pseudo-first order curves.

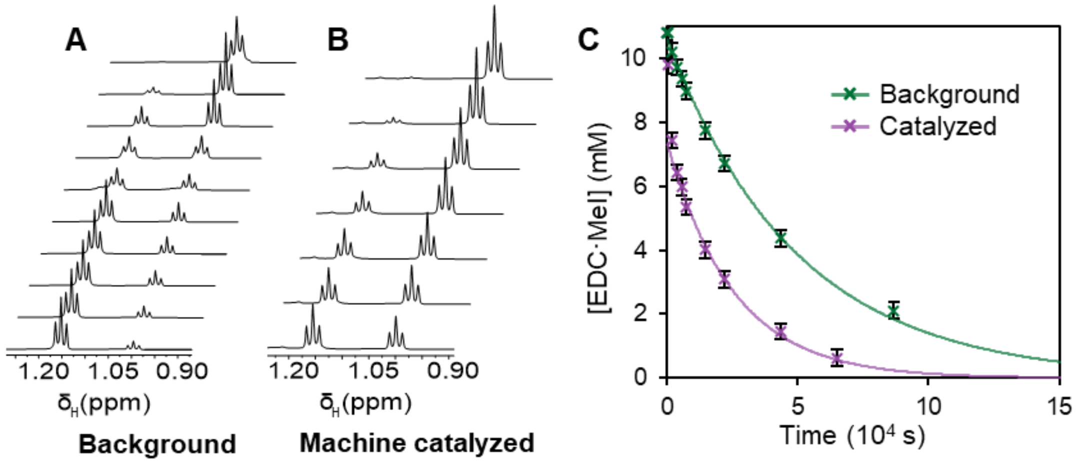

Figure S3 Kinetics of EDC·MeI hydration with a HOBt additive at pH_{obs} 5.36 in CD_3CN/D_2O (7:3 *v*/*v*). Stack-plots of partial ^1H NMR spectra following the background (**A**) and machine catalyzed (**B**) hydration of EDC·MeI. (**C**) The plotted data derived from the ^1H NMR spectra with the fitted pseudo-first order curves.

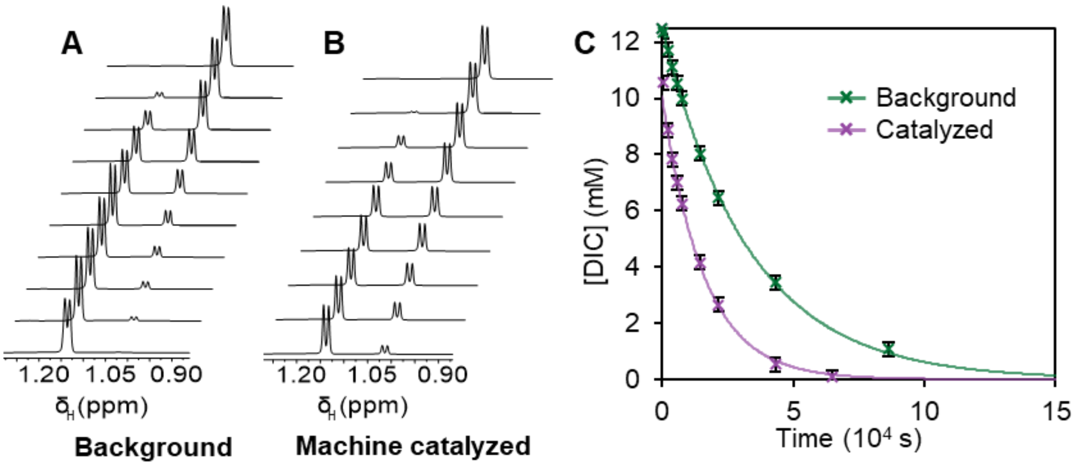

Figure S4 Kinetics of DIC hydration with a HOAt additive at pH_{obs} 5.36 in 7:3 CD_3CN/D_2O (*v*/*v*). Stack-plots of partial ^1H NMR spectra following the background (**A**) and machine catalyzed (**B**) hydration of DIC. (**C**) The plotted data derived from the ^1H NMR spectra with the fitted pseudo-first order curves.

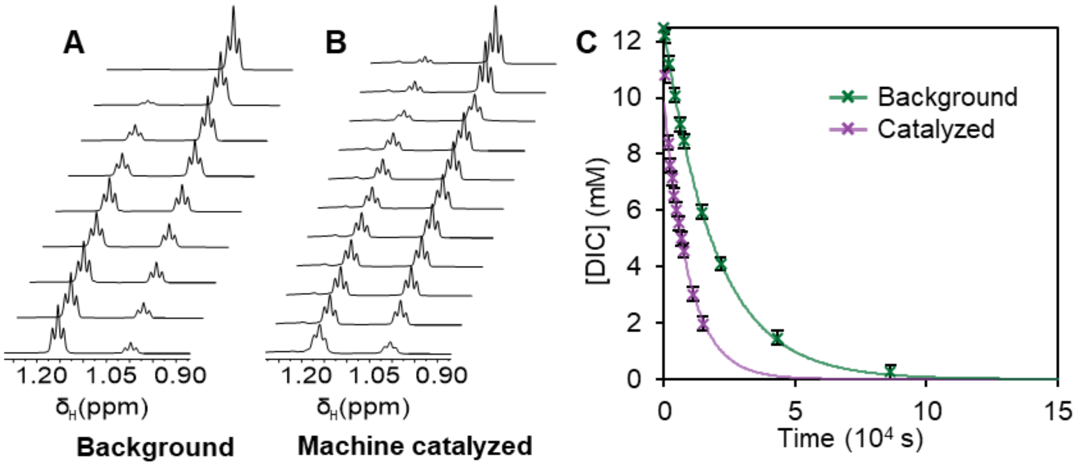

Figure S5 Kinetics of EDC·MeI hydration with a HOAt additive at pH_{obs} 5.36 in CD_3CN/D_2O (7:3 *v*/*v*). Stack-plots of partial ^1H NMR spectra following the background (**A**) and machine catalyzed (**B**) hydration of DIC. (**C**) The plotted data derived from the ^1H NMR spectra with the fitted pseudo-first order curves.

S3.1 General method for determining machine catalyzed rate

The rate of carbodiimide hydration observed in the ^{1}H NMR monitoring experiments is the linear combination of the rates of the machine catalyzed pathway and the background reaction, both of which can be approximated as pseudo-first order reactions under the reaction conditions (with a large excess of water) and when ratchet **1** has reached an approximately steady state during operation:

$$\frac{d[\mathrm{Fuel}]}{dt} \approx k_{\mathrm{obs}}[\mathrm{Fuel}] = k_{\mathrm{cat}}[\mathrm{Fuel}] + k_{\mathrm{uncat}}[\mathrm{Fuel}] \qquad \textbf{equation S1}$$

where k_{obs} is the apparent pseudo-first order rate constant for the reaction in the presence of ratchet **1**, k_{cat} is the pseudo-first order rate constant for only the machine catalyzed pathway and k_{uncat} is the rate constant for the background reaction. To find the rate of the catalyzed reaction, k_{uncat} can be measured independently by observation of the rate in the absence of the machine catalyst, so k_{cat} can be simply computed as $k_{\mathrm{obs}} - k_{\mathrm{uncat}}$ (Table S1).

Table S1 Values for the pseudo-first order rate constants for carbodiimide hydration reactions in CD_3CN/D_2O (7:3 *v*/*v*) buffered to pH_{obs} 5.36 (measured in D_2O) with 2.5 mM ratchet **1** and 5.0 mM additive. The uncatalyzed rates $\boldsymbol{k}_{\mathrm{uncat}}$ are measured from background reactions with no ratchet **1** present, allowing $\boldsymbol{k}_{\mathrm{cat}}$ to be computed as $\boldsymbol{k}_{\mathrm{obs}} - \boldsymbol{k}_{\mathrm{uncat}}$.

Fuel	Additive	k_{obs} (× 10^{-5} s^{-1})	k_{uncat} (× 10^{-5} s^{-1})	k_{cat} (× 10^{-5} s^{-1})
DIC	HOBt	2.05 ± 0.01	1.20 ± 0.003	0.85 ± 0.01
DCC	HOBt	1.76 ± 0.03	1.02 ± 0.01	0.74 ± 0.03
EDC·MeI	HOBt	3.93 ± 0.02	2.02 ± 0.02	1.91 ± 0.03
DIC	HOAt	6.28 ± 0.01	3.01 ± 0.01	3.27 ± 0.01
EDC·MeI	HOAt	10.45 ± 0.06	5.09 ± 0.01	5.37 ± 0.06

S3.2 Determination of catalytic efficiencies

Catalytic efficiency can be calculated in two ways; considering the steady state case only or considering the proportion of fuel that reacts via the machine catalyzed pathway under the experimental conditions (Table S2). At the steady state, the catalytic efficiency ($\mathrm{C.E.}_{\mathrm{SS}}$) is given by the proportion of the observed rate during the machine catalyzed reaction that results from the machine catalyzed pathway,

$$\mathrm{C.E._{SS}} = \frac{k_{\mathrm{cat}}}{k_{\mathrm{obs}}}$$ **equation S2**

Alternatively, the proportion of fuel that reacts via the machine catalyzed pathway in the monitored reactions ($\mathrm{C.E._R}$) can be found by integrating the normalized difference between the pseudo-first order curves fitted for the catalyzed and uncatalyzed fuel-to-waste reactions:

$$\mathrm{C.E._R} = \frac{\left(\frac{[\mathrm{Fuel}]_0^{\mathrm{uncat}}}{k_{\mathrm{uncat}}} - \frac{[\mathrm{Fuel}]_0^{\mathrm{cat}}}{k_{\mathrm{obs}}}\right)}{\left(\frac{[\mathrm{Fuel}]_0^{\mathrm{uncat}}}{k_{\mathrm{uncat}}}\right)}$$ **equation S3**

where $[\mathrm{Fuel}]_0^{\mathrm{uncat}}$ is the initial concentration of the carbodiimide fuel estimated by the fitted equation for the uncatalyzed background reaction, while $[\mathrm{Fuel}]_0^{\mathrm{cat}}$ is the estimated initial fuel concentration during the catalysed reaction. As the curve is fitted based only on the steady state rates, the latter value underestimates the concentration of carbodiimide added to the reaction, thus taking account of the fuel used rapidly to enable the steady state to be reached.

Table S2 Values for the catalytic efficiency of ratchet **1** for carbodiimide hydration reactions in CD_3CN/D_2O (7:3 *v*/*v*) buffered to pH_{obs} 5.36 (measured in D_2O) with 2.5 mM ratchet **1** and 5.0 mM additive. $\mathrm{C.E._R}$ = catalytic efficiency of the reaction (see equation S3), $\mathrm{C.E._{SS}}$ = catalytic efficiency at steady state (see equation S2).

Fuel	Additive	$\mathrm{C.E._R}$	$\mathrm{C.E._{SS}}$
DIC	HOBt	52% ± 0.3%	41% ± 0.6%
DCC	HOBt	57% ± 1.1%	42% ± 1.7%
EDC·MeI	HOBt	64% ± 0.7%	49% ± 0.8%
DIC	HOAt	61% ± 0.2%	52% ± 0.2%
EDC·MeI	HOAt	61% ± 0.3%	51% ± 0.6%

Catalytic efficiency accounts for only part of the overall efficiency of a motor. The remaining portion is dependent on how effectively a ratchet uses the energy from the fuel-to-waste reactions that it catalyzes, termed the thermodynamic efficiency of a ratchet.[S2] While we do not measure this here, we

can find the related coefficient of performance that shows how many forward cycles a ratchet could be expected to achieve per molecule of fuel it uses in Section S4.3.

S4. Measurement of directionality

The directionality of the system can be measured as a ratio of the distal and proximal co-conformers of rotaxane **1′**, which can be trapped out as rotaxane **2** by the formation of an amide to prevent further reaction. The amine can be added at different times after the fuel to trap the ratchet out at different points during the operation[S1,S3] (main text, Figure 1). After work up, the percentage of *prox*-**2** can be assessed by integration of ^{1}H NMR peaks corresponding to the macrocycle-bound fumaramide compared to signals from the macrocycle (Figure S6). The change in the percentage of *prox*-**1′** over time (as determined by the percentage of *prox*-**2** when the operation is trapped at a given time) can be used to fit exponential decay equation (Figures S7–S11) of the form

$$\frac{[prox\text{-}\mathbf{1'}]}{[\mathbf{1}]_0} = \left(\mathrm{I}_{prox} - \mathrm{SS}_{prox}\right)e^{-k_r t} + \mathrm{SS}_{prox} \qquad \textbf{equation S4}$$

where $[prox\text{-}\mathbf{1}']/[\mathbf{1}]_0$ is the proportion of ratchet **1** that exists as *prox*-**1′** at any given time during operation. I_{prox} is the initial proportion of *prox*-**1′** after the first barrier formation, SS_{prox} is the steady state proportion of *prox*-**1′** under the fueling conditions, k_r is a rate constant describing the rate at which the ratchet progresses towards the steady state and t is the time in seconds since the fuel was added.

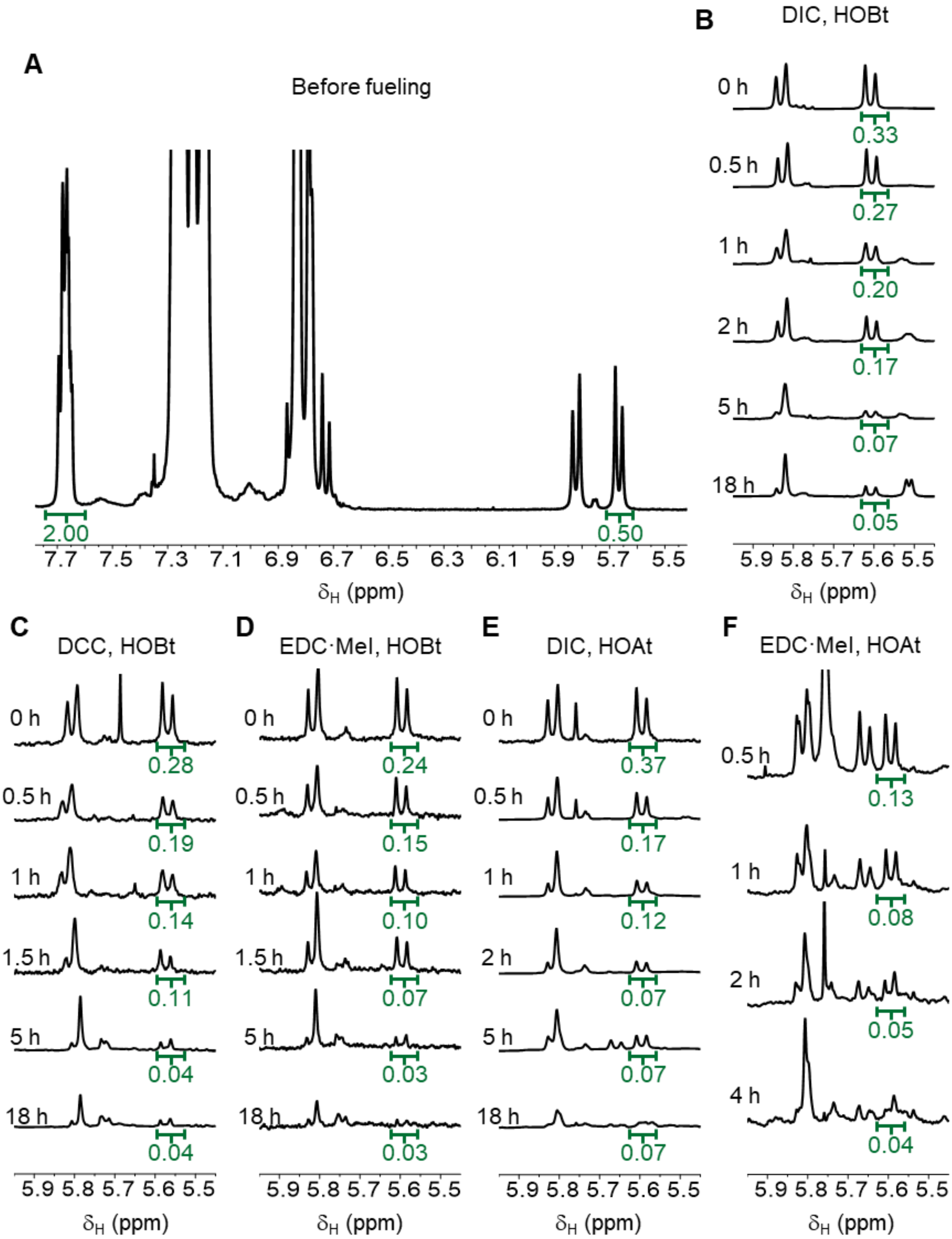

Figure S6 (**A**) Partial ^{1}H NMR spectrum of the unfueled ratchet **1** showing the peak used for integral normalization at 7.65 ppm, corresponding to two macrocycle protons (regardless of macrocycle position), and peaks corresponding to the macrocycle-bound protonated fumaramide station, indicating the proportion of rotaxane **1** in the proximal conformation. (**B**–**F**) Partial ^{1}H NMR spectra showing the peaks indicative of *prox*-**2**, formed when for ratchet **1** is fueled with the indicated carbodiimide and additive before being trapped by amide formation at the set time. For some mixtures (**F**), there is unreacted **1** remaining in the mixture after trapping due to relatively higher amounts of **1** compared to **1′** in the steady state: this is accounted for when assessing the relative proportions.

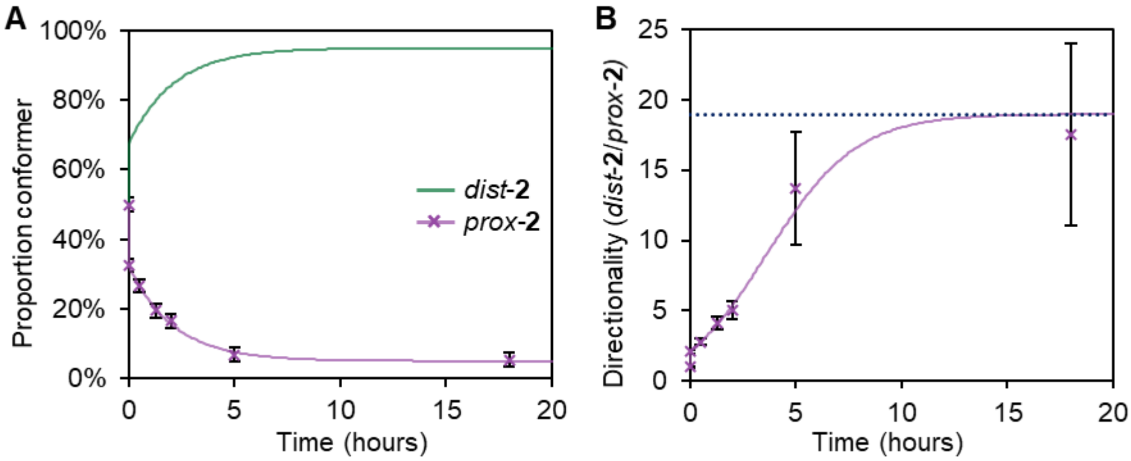

Figure S7 The directionality ratchet **1** with DIC fuel and HOBt additive. (**A**) The measured proportion of *prox*-**2** and *dist*-**2**, as determined from the ^{1}H NMR spectra show in Figure S6B, are used to fit an exponential decay to a steady state (equation S4). (**B**) The directionality can then be determined as a ratio, where the steady state directionality is indicated by the dotted blue line.

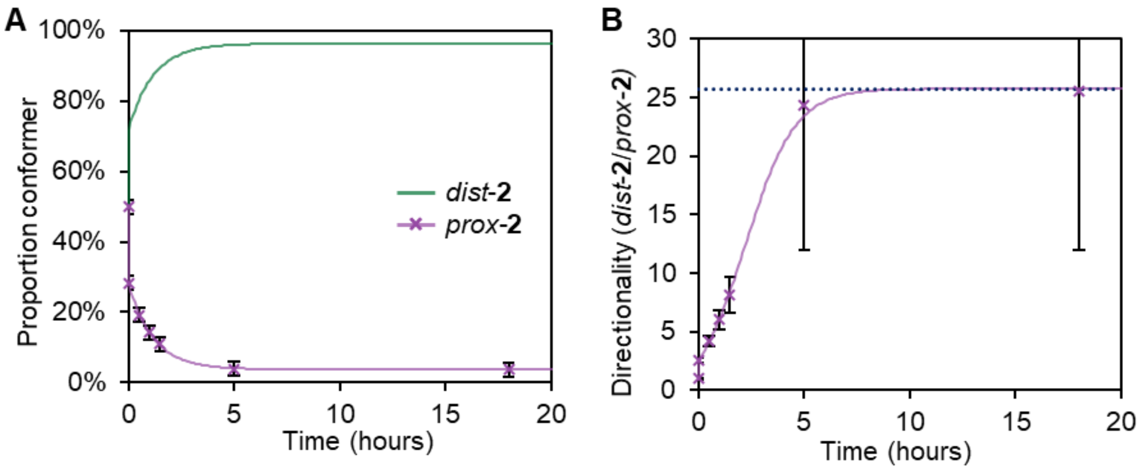

Figure S8 The directionality ratchet **1** with DCC fuel and HOBt additive. (**A**) The measured proportion of *prox*-**2** and *dist*-**2**, as determined from the ^{1}H NMR spectra show in Figure S6C, are used to fit an exponential decay to a steady state (equation S4). (**B**) The directionality can then be determined as a ratio, where the steady state directionality is indicated by the dotted blue line.

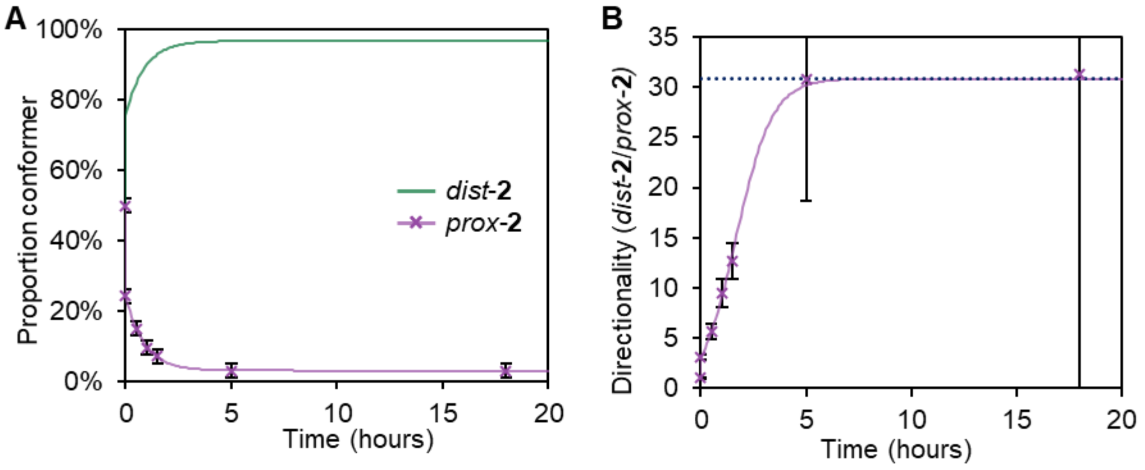

Figure S9 The directionality ratchet **1** with EDC·MeI fuel and HOBt additive. (**A**) The measured proportion of *prox*-**2** and *dist*-**2**, as determined from the ^{1}H NMR spectra show in Figure S6D, are used to fit an exponential decay to a steady state (equation S4). (**B**) The directionality can then be determined as a ratio, where the steady state directionality is indicated by the dotted blue line.

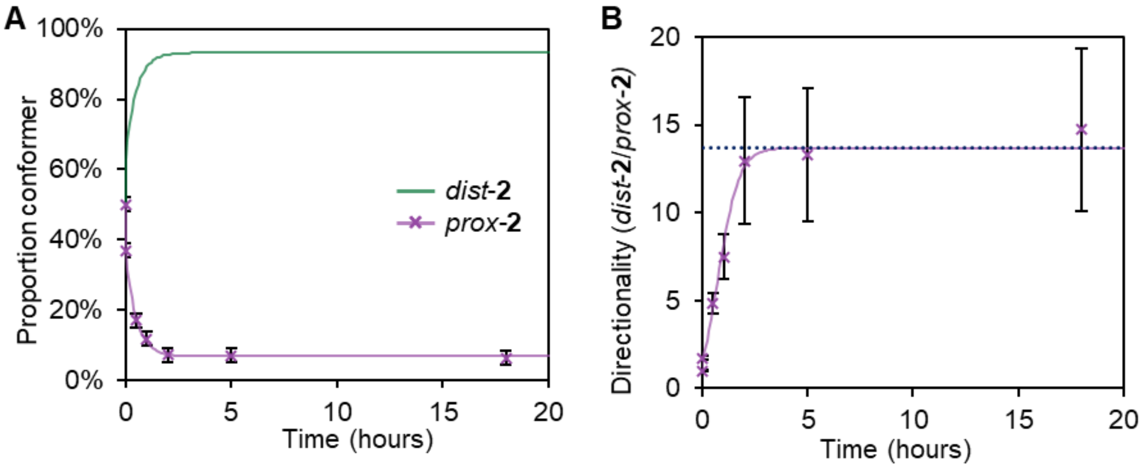

Figure S10 The directionality ratchet **1** with DIC fuel and HOAt additive. (**A**) The measured proportion of *prox*-**2** and *dist*-**2**, as determined from the ^{1}H NMR spectra show in Figure S6E, are used to fit an exponential decay to a steady state (equation S4). (**B**) The directionality can then be determined as a ratio, where the steady state directionality is indicated by the dotted blue line.

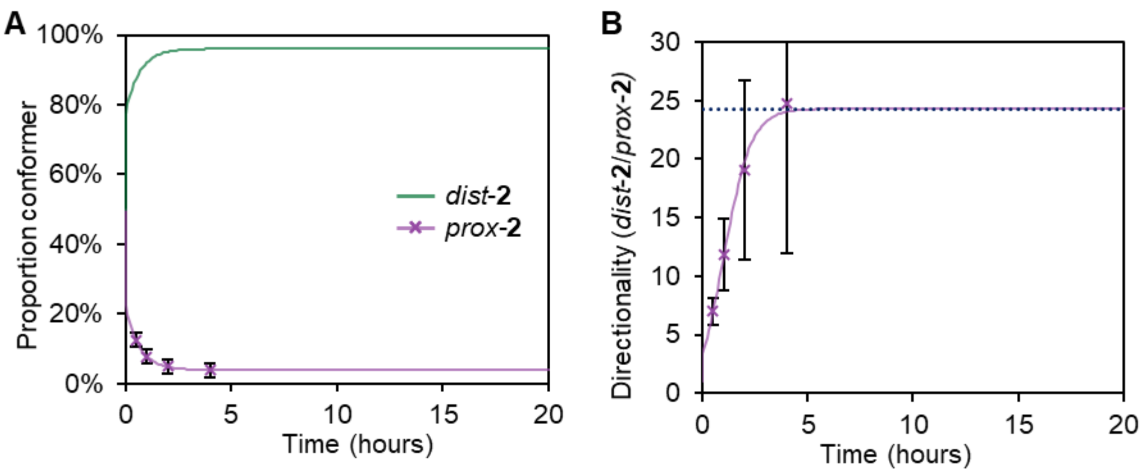

Figure S11 The directionality ratchet **1** with EDC·MeI fuel and HOAt additive. (**A**) The measured proportion of *prox*-**2** and *dist*-**2**, as determined from the ^{1}H NMR spectra show in Figure S6F, are used to fit an exponential decay to a steady state (equation S4). (**B**) The directionality can then be determined as a ratio, where the steady state directionality is indicated by the dotted blue line.

S4.1 The gating of ester formation and hydrolysis

The ratio of the steady state populations of *dist*-**1′** and *prox*-**1′** (SS_{dist}/SS_{prox}) is the overall directionality of the ratchet (Table S3) and also provides a direct measure of kinetic asymmetry via the ratcheting constant K_r see (Section S4.2). For this ratchet, with no power stroke component,[S4–S8] the directionality is the product of the kinetic gating of the two chemical steps: formation and hydrolysis of the activated ester. The gating of the formation can be found directly if the ratio of *dist*-**1′** and *prox*-**1′** is trapped out (before any hydrolysis can occur) by the addition of the amine trapping reagent, *p*-$MeOC_6H_4NH_2$, before the addition of the fuel. In equation S4 this is represented by the value of $[\mathrm{I}_{prox}]$, where the gating of the ester formation step would be given by

$$\text{Formation gating} = \frac{\mathrm{I}_{dist}}{\mathrm{I}_{prox}} = \frac{(1-\mathrm{I}_{prox})}{\mathrm{I}_{prox}} \qquad \textbf{equation S5}$$

Any additional directionality must arise from gating of the ester hydrolysis, allowing this to be found by dividing the overall directionality by the directionality due to the ester formation

$$\text{Hydrolysis gating} = \frac{\mathrm{I}_{dist}\,\mathrm{SS}_{prox}}{\mathrm{I}_{prox}\,\mathrm{SS}_{dist}} = \frac{(1-\mathrm{I}_{prox})\mathrm{SS}_{prox}}{\mathrm{I}_{prox}(1-\mathrm{SS}_{prox})} \qquad \textbf{equation S6}$$

Table S3 Values for the overall directionality, ester formation gating and ester hydrolysis gating for ratchet **1** when fueled by carbodiimide hydration reactions in CD_3CN/D_2O (7:3 *v*/*v*) buffered to pH_{obs} 5.36 (measured in D_2O) with 2.5 mM ratchet **1** and 5.0 mM additive.

Fuel	Additive	Overall Directionality SS_{dist}/SS_{prox}	Formation gating I_{dist}/I_{prox}	Hydrolysis gating $I_{dist}\,SS_{prox}/I_{prox}\,SS_{dist}$
DIC	HOBt	20.3 ± 2.0	2.2 ± 0.03	9.3 ± 0.9
DCC	HOBt	25.7 ± 2.7	2.6 ± 0.04	9.4 ± 1.1
EDC·MeI	HOBt	30.9 ± 2.9	3.1 ± 0.04	9.7 ± 0.9
DIC	HOAt	13.7 ± 1.5	1.7 ± 0.04	8.0 ± 0.9
EDC·MeI	HOAt	24.3 ± 1.1	3.5 ± 0.03	7.0 ± 0.3

S4.2 Equating directionality and the ratcheting constant (kinetic asymmetry)

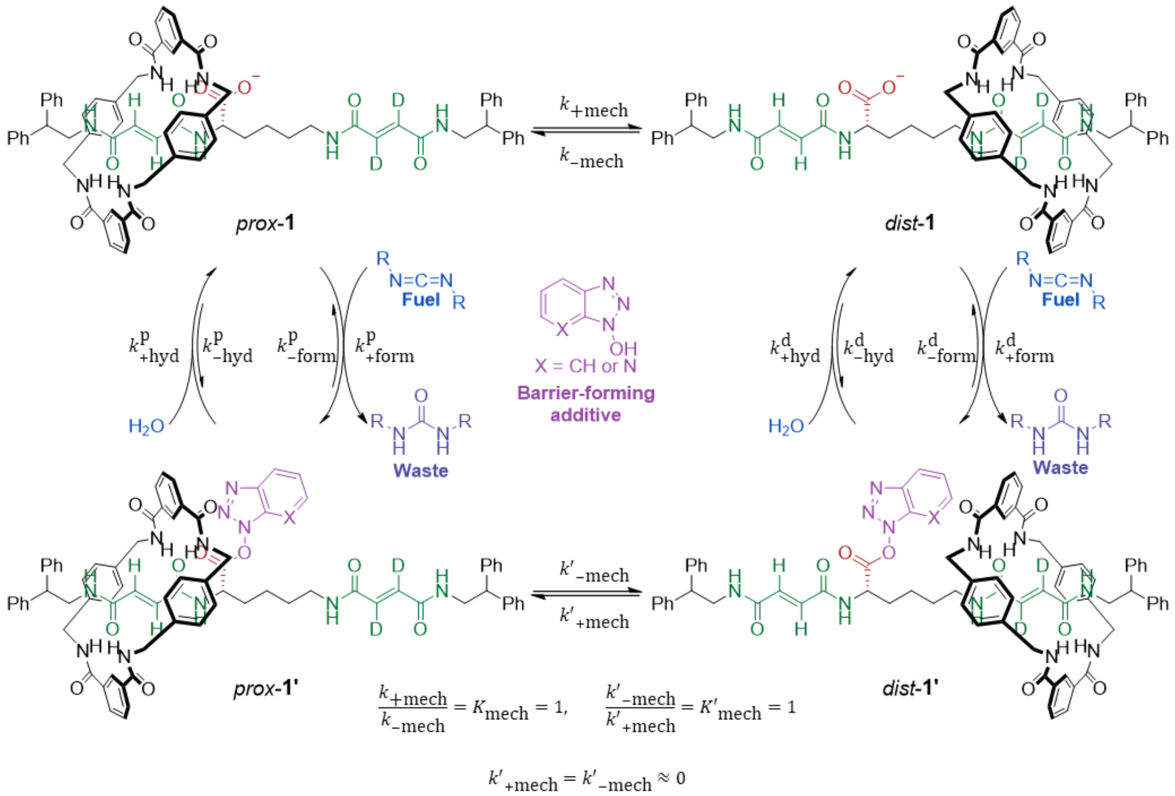

$$\frac{k_{+mech}}{k_{-mech}} = K_{mech} = 1, \quad \frac{k'_{-mech}}{k'_{+mech}} = K'_{mech} = 1$$

$$k'_{+mech} = k'_{-mech} \approx 0$$

Figure S12 A chemomechanical for the operation of ratchet **1**, with each transition labelled. In this ratchet, kinetic asymmetry is introduced by chemical gating only, while the mechanical transitions do not introduce bias.

Kinetic asymmetry is an essential feature of information ratchet mechanisms[S4–S8] as it is a prerequisite for ratchets to transduce energy from a fuel-to-waste reaction to perform work.[S2,S9] The ratcheting constant (K_r) is a way of expressing kinetic asymmetry in a form similar to an equilibrium constant, though it expresses how far a ratcheted process can be driven away from equilibrium.[S4] K_r for this ratchet can be expressed as

$$K_r = \left(\frac{k^{\mathrm{p}}_{+\mathrm{hyd}}+k^{\mathrm{p}}_{-\mathrm{form}}}{k^{\mathrm{p}}_{-\mathrm{hyd}}+k^{\mathrm{p}}_{+\mathrm{form}}}\right) K_{\mathrm{mech}} \left(\frac{k^{\mathrm{d}}_{-\mathrm{hyd}}+k^{\mathrm{d}}_{+\mathrm{form}}}{k^{\mathrm{d}}_{+\mathrm{hyd}}+k^{\mathrm{d}}_{-\mathrm{form}}}\right) K'_{\mathrm{mech}} \qquad \textbf{equation S7}$$

with reference to Figure S12, where K_{mech} and K'_{mech} are equilibrium constants for mechanical exchange between the binding sites respectively with or without the barrier in place and are both taken to be 1 for this system as the binding sites are degenerate and both coordinate the macrocycle with equal strength (although the presence of the barrier and the stoppers make the rate of equilibration with the barrier in place effectively zero). This is an assumption on part of K'_{mech} as this cannot be measured due to the slow exchange. However as there are both interactions that might weaken macrocycle binding in the proximal coconformer (e.g., steric clash and restricted ester rotation) and interactions that might strengthen macrocycle binding (e.g., π-π stacking and hydrogen bonding), this seemed the most reasonable approximation. k denotes a rate constant for a reaction. The superscript represents whether the reaction concerned occurs with the macrocycle is proximal (p) or distal (d) to the reactive site, hydrolysis reactions are denoted with a subscript hyd while subscript form indicates the carbodiimide induced active ester formation. A +-symbol shows the reaction progresses in the thermodynamically favored direction, while a −-symbol represents the microscopic reverse of the process (i.e., spontaneous formation of the active ester as the reverse of hydrolysis, or the dehydration of urea waste to hydrolyze an active ester). The rate constants for the microscopic reverse processes for this system are likely to be several orders of magnitude smaller than the forwards processes so are unlikely to play a significant role, although they are included in the analysis for completeness. Either by approximating the reverse processes as negligible, or by combining the chemical transformations with the same effect into a single rate constant (e.g., $k^{\mathrm{p}}_{+\mathrm{hyd}} + k^{\mathrm{p}}_{-\mathrm{form}} = k^{\mathrm{p}}_{\mathbf{1'}\to\mathbf{1}}$), the expression for K_r can be simplified to

$$K_r = \left(\frac{k^{\mathrm{p}}_{\mathbf{1'}\to\mathbf{1}}}{k^{\mathrm{p}}_{\mathbf{1}\to\mathbf{1'}}}\right)\left(\frac{k^{\mathrm{d}}_{\mathbf{1}\to\mathbf{1'}}}{k^{\mathrm{d}}_{\mathbf{1'}\to\mathbf{1}}}\right) = \overbrace{\frac{k^{\mathrm{d}}_{\mathbf{1}\to\mathbf{1'}}}{k^{\mathrm{p}}_{\mathbf{1}\to\mathbf{1'}}}}^{\substack{\text{Formation}\\\text{gating}}} \; \overbrace{\frac{k^{\mathrm{p}}_{\mathbf{1'}\to\mathbf{1}}}{k^{\mathrm{d}}_{\mathbf{1'}\to\mathbf{1}}}}^{\substack{\text{Hydrolysis}\\\text{gating}}} = \text{Overall directionality} \qquad \textbf{equation S8}$$

Therefore, the steady state directionality measured by the trapping experiments acts as a direct observation of the ratcheting constant and kinetic asymmetry for this ratchet.

Interestingly, under the conditions where at least one mechanical transition in a chemical engine cycle is slow, such as in the present system, the ratcheting constant is also indicative of the maximum amount of energy can be stored in the ratcheted system while it is maintained at a steady state away from equilibrium.[S4,S10] Therefore, the directionality can be directly related to the amount of work the ratchet performs to pump the macrocycle away from the equilibrium distribution.

$$\text{Ratcheted work} = RT \ln K_r \qquad \textbf{equation S9}$$

S4.3 Fuel molecules used per forward step

From the gating of the individual chemical steps, it is possible to work out how many forward steps the ratchet will achieve per unit of fuel that reacts with the machine (Table S4). This functions as a coefficient of performance for a theoretical motor based on the ratchet which allows comparison between different motor designs. This coefficient of performance is related to the thermodynamic efficiency of the ratchet,[S2,S9] though it is independent of the free energy released by the specific fuel-to-waste reaction used and the amount of work performed by the ratchet. To calculate the coefficient of performance the probabilities of each pathway round the ratchet's chemomechanical cycle are considered, along with the contribution to forwards motion:

$$\text{Coefficient of performance (C.O.P.)} = \overbrace{\left(\frac{\text{F.G.}}{\text{F.G.}+1}\right)\left(\frac{\text{H.G.}}{\text{H.G.}+1}\right)}^{\text{Complete forward cycle}} - \overbrace{\left(\frac{1}{\text{F.G.}+1}\right)\left(\frac{1}{\text{H.G.}+1}\right)}^{\text{Complete backwards cycle}} \qquad \textbf{equation S10}$$

The quantities of formation gating (F.G.) and hydrolysis gating (H.G.) represent the ratio of the forward chemical transformation and the corresponding backward chemical transformation, with the latter normalized as one. Therefore, the probability of a chemical transformation can be represented as in equation *(S10)*. Naturally, the completion of a full forwards cycle has a positive contribution, using one molecule of fuel to take a step, while the completion of a backwards cycle has a negative contribution as a fuel molecule is hydrated and a backwards step is taken. Futile cycles, in which a fuel molecule is used but not step is taken, make up the remaining probability. Futile cycles necessitate the consideration of the gating of individual chemical transitions, and not just the overall directionality, when considering the efficiency of a motor. Motors with only one chemically gated transition are limited to a maximum of 50% of fuel used leading to a forward cycle, not because of backward cycles, but because at least half the reactions with fuel will result in a futile cycle because of the ungated chemical transition. Therefore, a motor with double chemical gating, with a bias of 2:1 for both transitions, will be more efficient than a singly chemically gated motor with a 4:1 bias, even though both motors will achieve the

same ultimate directional bias. It is more efficient to have a machine where the introduction of directional bias is distributed across the chemical engine cycle: this observation also arises when considering the thermodynamics of these systems and studying how they most efficiently dissipate energy.[S9,S11–S13]

The product of the coefficient of performance and the catalytic efficiency of the motor (Section S3.2), can be used as a good measure of the effectiveness with which a ratchet uses fuel to move directionally (Table S4). This is closely related to the overall efficiency with which the ratchet performs work, although this also depends on the energy released by the fuel-to-waste reaction and the force against which a ratchet is working. Neither of these is standardized under these conditions: the former changes with relative concentration of the fuel and waste, while the latter depends on the average distribution (or probability distribution) of the macrocycle.

Table S4 Values for the coefficient of performance and hence the percentage of steps taken per molecule of fuel added to the reaction for ratchet **1** fueled by carbodiimide hydration reactions in CD_3CN/D_2O (7:3 *v*/*v*) buffered to pH_{obs} 5.36 (measured in D_2O) with 2.5 mM ratchet **1** and 5.0 mM additive. C.O.P. = coefficient of performance, see equation S10.

Fuel	Additive	C.O.P.	Steps per fuel
DIC	HOBt	58% ± 18%	30% ± 9%
DCC	HOBt	62% ± 14%	36% ± 8%
EDC·MeI	HOBt	66% ± 13%	43% ± 8%
DIC	HOAt	52% ± 12%	32% ± 7%
EDC·MeI	HOAt	65% ± 6%	40% ± 4%

S5. Net rate of forward movement

Once the rate of the machine catalyzed reaction (see Section S3.1) and the number of forward cycles achieved per unit of fuel (see Section S4.3) are known, the rate of forward cycling, the speed of a theoretical motor, can be derived.

$$\text{Speed (s}^{-1}) = (k_{\text{cat}} \times \text{C.O.P.}) \frac{[\text{Fuel}]}{[\mathbf{1}]_0} \qquad \textbf{equation S11}$$

k_{cat} is the pseudo-first order rate constant for the catalyzed reaction and the coefficient of performance (C.O.P.) is the net number of forward rotations achieved per unit of fuel, $[\text{Fuel}]$ is the concentration of carbodiimide present in the reaction, and $[\mathbf{1}]$ is the total concentration of the ratchet in the reaction. We note that this model is valid only where the pseudo-first order approximation is an appropriate estimation of the rate of fuel use. For higher fuel concentrations, a more detailed description of the reaction network is required.[S9,S14] However, as all operations fall within this range, we suggest the most useful representation of the speed for comparison between motor designs is achieved by taking the cycle rate per hour at a set concentration of fuel (e.g., mM^{-1}):

$$\frac{\text{Speed}\ (\text{h}^{-1})}{[\text{Fuel}]} = \frac{(k_{\text{cat}} \times \text{C.O.P.})}{[\mathbf{1}]_0} \times 3600\ \text{s}\ \text{h}^{-1} \qquad \textbf{equation S12}$$

Table S5 Values for the rate of steps or rotation that would be achieved by a molecular motor based on ratchet **1** per mM available fuel when fueled by carbodiimide hydration reactions in CD_3CN/D_2O (7:3 *v*/*v*) buffered to pH_{obs} 5.36 (measured in D_2O) with 2.5 mM ratchet **1** and 5.0 mM additive.

Fuel	Additive	Speed (× 10^2 h^{-1} mM^{-1})
DIC	HOBt	0.70 ± 0.22
DCC	HOBt	0.67 ± 0.15
EDC·MeI	HOBt	1.83 ± 0.36
DIC	HOAt	2.45 ± 0.56
EDC·MeI	HOAt	5.03 ± 0.46

S6. Comment on errors

All errors were propagated from the standard deviations obtained for the fitted curves of the rate of fuel use and the directionality experiments. Due to the limitations of bulk NMR methods used in the present study (obtaining ratio of a very small peak relative to a big peak), some of the errors are too large to draw significant conclusions when comparing some similar results. We anticipate that single molecule techniques may be required to obtain more accurate assessments of the values.

S7. X-ray crystal structure information

Data Collection. X-Ray data for compound **1** were collected at 100 K on a Rigaku FR-X rotating anode equipped with Hypix-6000HE detectors and oxford cryosystem. Data were measured using CrysAlisPro suite of programs.

Crystal structure determination and refinement. X-Ray data were processed and reduced using CrysAlisPro suite of programmes. Absorption correction was performed using empirical methods (SCALE3 ABSPACK) based upon symmetry-equivalent reflections combined with measurements at different azimuthal angles. The crystal structures were solved and refined against all F^2 values using the SHELX and Olex 2 suite of programmes.[S15–16] Crystal structure of **1** presented some disorder in the macrocycle phenyl groups and the MEOH and DCM solvent molecules. The disorder was modelled over two positions, where atomic distances were restrained using distance restrains (SHELX; DFIX and SADI). The atomic displacement parameters (adp) were restrained using rigid body restrains (SHELX RIGU and SIMU commands). Hydrogen atoms were placed in the calculated positions.

CCDC 2191007 contains the supplementary crystallographic data for this paper. These data can be obtained free of charge via www.ccdc.cam.ac.uk/conts/retrieving.html (or from the Cambridge Crystallographic Data Centre, 12 Union Road, Cambridge CB21EZ, UK; fax: (+44)1223-336-033; or deposit@ccdc.cam.ac.uk).

Table S6. Crystallographic parameters

Identification code	**1**
Empirical formula	$C_{82}H_{92}ClD_2N_8O_{13}$
Formula weight	1437.11
Temperature/K	99.96(12)
Crystal system	monoclinic
Space group	$P2_1$
a/Å	14.6517(4)
b/Å	9.7689(3)
c/Å	26.4885(9)
α/°	90
β/°	92.141(3)
γ/°	90
Volume/Å^3	3788.7(2)
Z	2
ρ_{calc}g/cm^3	1.260
μ/mm^{-1}	1.003
F(000)	1526.0
Crystal size/mm^3	0.03 × 0.03 × 0.01
Radiation	CuKα (λ = 1.54184)
2Θ range for data collection/°	6.036 to 153.608
Index ranges	−16 ≤ h ≤ 17, −12 ≤ k ≤ 12, −33 ≤ l ≤ 32

Reflections collected	46913
Independent reflections	15040 [R_{int} = 0.0455, R_{sigma} = 0.0517]
Data/restraints/parameters	15040/239/1037
Goodness-of-fit on F^2	1.051
Final R indexes [I>=2σ (I)]	R_1 = 0.0572, wR_2 = 0.1470
Final R indexes [all data]	R_1 = 0.0649, wR_2 = 0.1535
Largest diff. peak/hole / e Å^{-3}	0.52/−0.68
Flack parameter	0.041(18)

S8. References

S1 Borsley, S.; Leigh, D. A.; Roberts, B. M. W. A doubly kinetically-gated information ratchet autonomously driven by carbodiimide hydration. *J. Am. Chem. Soc.* **2021**, *143*, 4414–4420.

S2 Borsley, S.; Leigh, D. A.; Roberts, B. M. W. Chemical fuels for molecular machinery. *Nat. Chem.* **2022**, *14*, 728–738.

S3 Schnitter, F.; Boekhoven, J. A method to quench carbodiimide-fueled self-assembly. *ChemSystemsChem* **2021**, *3*, e2000037.

S4 Ragazzon, G.; Prins, L. J. Energy consumption in chemical fuel-driven self-assembly. *Nat. Nanotechnol.* **2018**, *13*, 882–889.

S5 Das, K.; Gabrielli, L.; Prins, L. J. Chemically fueled self-assembly in biology and chemistry. *Angew. Chem. Int. Ed.* **2021**, 60, 20120–20143.

S6 Astumian, R. D. Kinetic asymmetry allows macromolecular catalysts to drive an information ratchet. *Nat. Commun.* **2019**, *10*, 3837.

S7 Astumian, R. D. Irrelevance of the power stroke for the directionality, stopping force, and optimal efficiency of chemically driven molecular machines. *Biophys. J.* **2015**, *108*, 291–303.

S8 Astumian, R. D.; Mukherjee, S.; Warshel, A. The physics and physical chemistry of molecular machines. *ChemPhysChem* **2016**, *17*, 1719–1741.

S9 Amano, S.; Esposito, M.; Kreidt, E.; Leigh, D. A.; Penocchio, E. Roberts, B. M. W. Insights from an information thermodynamics analysis of a synthetic molecular motor. *Nat. Chem.* **2022**, *14*, 530–537.

S10 Penocchio, E.; Rao, R.; Esposito, M. Thermodynamic efficiency in dissipative chemistry. *Nat. Commun.* **2019**, *10*, 3865.

S11 Efremov, A.; Wang, Z. Universal optimal working cycles of molecular motors. *Phys. Chem. Chem. Phys.* **2011**, *13*, 6223–6233.

S12 Wagoner, J. A.; Dill, K. A. Mechanisms for achieving high speed and efficiency in biomolecular machines. *Proc. Natl. Acad. Sci. USA* **2019**, *116*, 5902–5907.

S13 Brown, A. I.; Sivak, D. A. Theory of nonequilibrium free energy transduction by molecular machines. *Chem. Rev.* **2020**, *120*, 434–459.

S14 Borsley, S.; Kredt, E.; Leigh, D. A.; Roberts, B. M. W. Autonomous chemically fuelled directional rotation about a single bond. *Nature* **2022**, *604*, 80–85.

S15 Sheldrick, G. M. Crystal structure refinement with SHELXL. *Acta Crystallogr. Sect. C Struct. Chem*. **2015**, *71*, 3–8.

S16 Dolomanov, O. V., Bourhis, L. J., Gildea, R. J., Howard, J. A. K. & Puschmann, H. OLEX2 : a complete structure solution, refinement and analysis program. *J. Appl. Crystallogr*. **2009**, *42*, 339–341.
